# The Increasing Age of TBI Patients at a Single Level 1 Trauma Center and the Discordance Between GCS and CT Rotterdam Scores in the Elderly

**DOI:** 10.3389/fneur.2020.00112

**Published:** 2020-02-20

**Authors:** Nicholas Garza, Atrin Toussi, Machelle Wilson, Kiarash Shahlaie, Ryan Martin

**Affiliations:** ^1^School of Medicine, University of California, Davis, Sacramento, CA, United States; ^2^Division of Biostatistics, Department of Public Health Sciences, University of California, Davis, Sacramento, CA, United States; ^3^Department of Neurological Surgery, University of California, Davis, Sacramento, CA, United States; ^4^Department of Neurology, University of California, Davis, Sacramento, CA, United States

**Keywords:** traumatic brain injury, TBI, geriatrics, Glasgow Coma Scale, CT Rotterdam Score, geriatric TBI

## Abstract

**Introduction:** Traumatic brain injury (TBI) is frequently encountered in geriatric patients, but there is a paucity of data describing TBI in the elderly. Here, we show the age of patients with TBI is increasing at our medical center and discuss the relationship between age and injury severity with patient outcomes.

**Methods:** This is a retrospective analysis of 3,179 adult patients with TBI treated at the University of California, Davis Level 1 Trauma Center between 2009 and 2016. Age, Glasgow Coma Scale (GCS), and CT Rotterdam Scores were recorded. Age was analyzed as both a continuous and categorical variable (18–34, 35–50, 51–65, >65 years-old). Extended Glasgow Outcome Scale was obtained at 3 and 6 months and dichotomized into favorable and unfavorable outcomes. Multivariable general linear regression models, chi-square, logistic regression analyses and ANOVA were used for statistical analyses; a *p* < 0.05 was considered significant.

**Results:** The mean age of patients was 52.2 ± 21.9 years with a male predominance (69%). There was a significant trend (*p* = 0.002) toward an increase in mean age each year, increasing by 4.4 years (*p* = 0.008) over the course of the analysis. Older patients had a higher mean GCS compared to younger patients with the same CT Rotterdam Score (*p* = 0.027), this becoming more pronounced with worse CT Rotterdam Scores. The >65 group had a 4-fold increased risk for unfavorable outcome when compared to the 18–34 group, this effect being most pronounced after mild TBI.

**Conclusions:** The mean age of TBI patients is increasing at our trauma center. The largest disparity in outcomes across age was seen in patients with a mild GCS and low CT Rotterdam Scores, suggesting that these markers of injury severity may underestimate the severity of injury in the elderly population. This information highlights the need for clinical trials and validation of outcome markers in geriatric TBI.

## Introduction

Traumatic brain injury (TBI) is a leading cause of morbidity and mortality worldwide. In the United States, TBI contributes to ~30% of deaths related to injury (1), and leads to 2.53 million ED visits, hospitalizations and deaths annually (2). Historically, motor vehicle crash (MVC) has been the leading cause of TBI in the United States, but analysis of recent demographic data highlights a growing proportion of fall-related injuries, surpassing MVCs as the leading cause of TBI in developed countries (1, 3). Similar trends have also been seen in Europe (4).

The major contributing factor to the changing incidence of TBI mechanisms is related to the growing elderly population. Falls are the most common mechanism of injury in the elderly secondary to the effects of age on physical function (5, 6). It is estimated that between 45 and 78% of geriatric patients are frail at the time of injury and this frailty has been associated with a 50% increase in mortality (7–10). One can reason, then, that the incidence of geriatric-related injuries will continue to increase as the elderly population continues to grow. In the United States, the population older than 65 years of age has increased from 12.4% in 2,000 to 14.9% in 2015 and this is expected to increase to 19.6% by 2030 (11, 12). In addition, the number of individuals over the age of 80 is predicted to increase by 10.2 million between 2000 and 2030 (12). Accordingly, the field of geriatric TBI will need to mature along with the aging population to better understand outcome prediction.

Differences in outcomes after TBI are associated with age, with outcomes worsening as early as age 45 (6, 13). While the incidence of TBI in the younger population has remained relatively unchanged, the incidence of TBI is increasing in the elderly population (10, 14, 15). In addition, while patients 65 years and older only account for 15% of the population, they make up 50% of TBI-related deaths (10, 12) and patients older than 75-years old experience twice the rate of TBI (10). This leads to increased resource utilization due to longer hospital stays and more frequent follow-up (6, 10, 13). In addition, acute measurements of injury severity, such as the Glasgow Coma Scale (GCS), may not be reliable predictors of morbidity and mortality in patients over the age of 45 (10). In one study by Livingstone et al. (13), even though older patients had less severe GCS scores, they had worse functional recovery when compared to younger populations.

Through a retrospective analysis of a prospectively maintained TBI registry at a Level 1 Adult Trauma Center, we show that the age of our patient population is increasing and discuss the relationship between age, severity of injury (both radiographic and clinical), and functional outcomes, and highlight the need for better predictive models in geriatric TBI.

## Methods

### Patients

This is a retrospective analysis of patient data collected from all adult patients with TBI that were treated at the University of California, Davis Level 1 Trauma Center between 2009 and 2016 as part of an Institutional TBI Registry. As a Level 1 Trauma Center in the United States, all patients, regardless of age, insurance status or ability to pay are stabilized, treated and if necessary, admitted per the Emergency Medical Treatment and Labor Act (EMTALA). Patients included in this registry were all patients seen by the neurological surgery service who met at least one of the following two criteria that prompted consultation: (1) suspected TBI due to clinical history, clinical symptoms, or signs of neurological deficits on physical examination, or (2) abnormal head computed tomography (CT) scan findings after trauma. Neurological surgery consultation is mandated at our institution for all TBI severities, as measured by the GCS. All patients 18-years or older were included in this study and baseline characteristics at the time of injury were obtained, including age, sex, mechanism of injury, and severity of injury. Age was grouped into four categories: 18–34, 35–50, 51–65, and older than 65 years.

### Mechanism and Severity of Injury

The mechanism of injury was recorded at the time of neurological surgery consultation and was categorized as assault, automobile vs. pedestrian (AvP), fall, motor vehicle crash (MVC), or “other.” Motor vehicle crashes included patients injured in an automobile or a motorcycle. Mechanisms of injury categorized as “other” included penetrating injuries, bicycle accidents, patients found down with no clear mechanism, fall from horse, fall from moving vehicle, sport related accident, or another mechanism not well-characterized. These other mechanisms each had too few incidences to allow for individual categorization. Clinical injury severity was recorded as the post-resuscitation GCS at the time of neurological surgery consultation and categorized as mild (GCS 13–15), moderate (GCS 9–12), or severe (GCS 3–8). The first CT head obtained after the TBI was used to calculate a CT Rotterdam Score, a commonly used measure of radiographic trauma severity that ranges from 1 (mild) to 6 (most severe) (16). Given our low sample sizes with a CT Rotterdam Score of 6, these categories were combined into the category 5 score for analysis.

### Outcomes

Trained research assistants performed phone interviews with either the patient or a surrogate to assess outcome, as measured by the Extended Glasgow Outcome Scale (GOSE). The GOSE is the most widely used measure of global functional outcome following TBI and has been recommended as the standard outcome measure for TBI studies (17, 18). Our primary outcome was a dichotomized GOSE (favorable vs. unfavorable) at three and 6 months. Patients with a favorable GOSE included lower moderate disability, upper moderate disability, lower good recovery, and upper good recovery, while unfavorable GOSE included upper severe disability, lower severe disability, vegetative, and dead.

### Statistical Analysis

Multivariable general linear regression models were used to examine the effects of age, CT Rotterdam Score, and GCS on the Glasgow Outcome Score. Chi-square analyses were used to examine relationships between categorical variables. Multivariable logistic regression was used to examine effects of age, CT Rotterdam Score, and GCS on categorical neurological outcomes (favorable or unfavorable) at 3 and 6 months. For logistic regression models, the age group “18–34” was used as the reference. ANOVA was used to examine relationships between categorical and continuous variables. A *p* of < 0.05 was considered significant. All statistical analyses were performed using SAS software v.9.4 (SAS Institute Inc., Cary, NC). Prior to analysis, approval was obtained from the UC Davis Institutional Review Board.

## Results

### Population Characteristics

**Figure 1:**
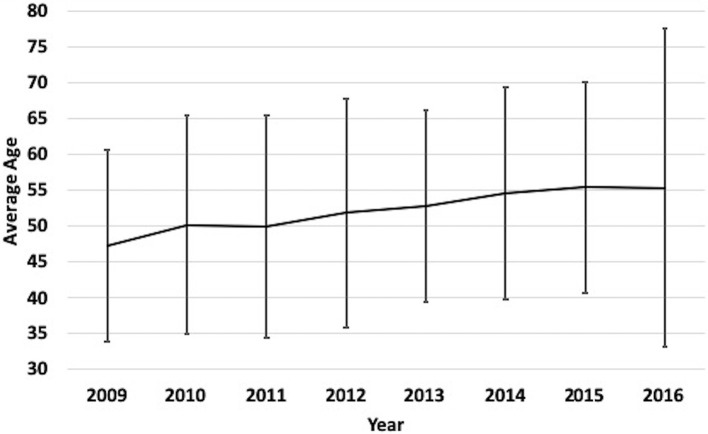
Yearly mean age of TBI patients at our medical Center from 2009 to 2016. Label: Bars indicate standard deviation.

A total of 3,179 patients were included in this analysis (Table 1). The mean age of the entire cohort was 52.2 ± 21.9 years and 69% were male. From 2009 to 2016, there was a significant trend (*p* = 0.002) toward an increase in average age each year, such that mean age increased by 4.4 years (*p* = 0.008) over the course of the analysis (Figure 1). Patients > 65 years of age was the most frequently encountered age group, representing 30% of all patients in the cohort, followed by the group aged 18–35 years (28%). Fall was the most frequent mechanism of injury (39.0%), an incidence more than double that of any of other mechanism (Table 1). The relationship between mechanism of injury and categorical age was significant (*p* = 0.008), such that patients who suffered a fall were nearly three times as likely to be in the oldest category when compared to other mechanisms of injury.

**Table 1:** Demographics of 3,179 traumatic brain injury patients seen at the University of California Davis Medical Center between 2009 and 2016.

	**Age group (years)**				
	**All**	**18–34**	**35–50**	**51–65**	**>65**
Number of patients (% total)	3,179 (100)	885 (28)	549 (17)	785 (25)	960 (30)
Age, years ± SD	52.2 ± 21.9	24.9 ± 4.7	43.4 ± 4.8	57.7 ± 4.2	79.0 ± 8.3
Gender (% male)	2207 (69)	717 (81)	470 (85)	505 (64)	515 (54)
GCS, *n* (%)^*^					
Mild	2221 (70)	534 (60)	356 (65)	557 (71)	774 (81)
Moderate	451 (14)	157 (18)	87 (16)	108 (14)	99 (10)
Severe	507 (16)	194 (22)	106 (19)	120 (15)	87 (9)
Mechanism of Injury, *n* (%)					
Assault	443 (14)	189 (21)	140 (23)	94 (13)	20 (2)
AvP	291 (9)	94 (11)	77 (13)	81 (11)	39 (4)
Fall	1241 (39)	109 (12)	143 (23)	267 (37)	722 (75)
MVC	629 (20)	300 (34)	113 (18)	130 (18)	86 (9)
Other	575 (18)	1933 (22)	142 (23)	147 (20)	93 (10)
CT Rotterdam Score *n* (%)	*n* = 1,969	*n* = 569	*n* = 349	*n* = 406	*n* = 645
1	76 (4)	36 (6)	11 (3)	15 (4)	14 (2)
2	841 (43)	236 (41)	157 (45)	164 (40)	284 (44)
3	744 (38)	176 (31)	112 (32)	171 (42)	285 (45)
4	183 (9)	67 (12)	44 (13)	31 (8)	41 (6)
5	97 (5)	34 (6)	19 (5)	23 (6)	21 (3)
6	28 (1)	20 (4)	6 (2)	2 (0)	0

AvP, auto vs. pedestrian; CT, computed tomography; GCS, Glasgow Coma Scale; MVC, motor vehicle crash; n, sample size; SD, standard deviation;

* *Differences in GCS noted across age groups (chi-square, p = 0.003)*.

### Severity of Injury

**Figure 2:**
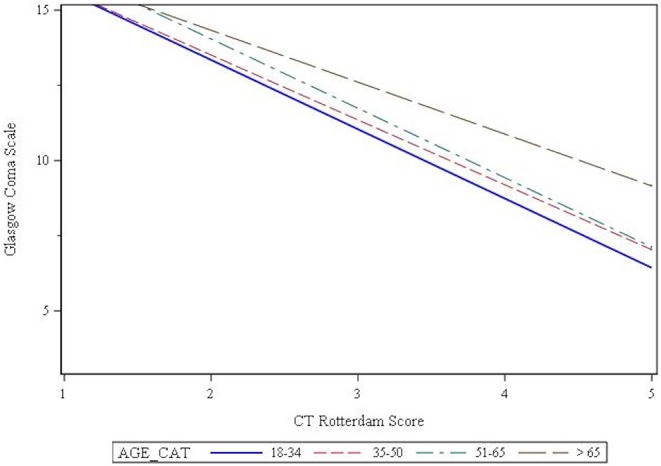
Interaction effects of age and CT Rotterdam Score on Glasgow Coma Scale.

Seventy percent of patients presented with a mild GCS, while 14 and 16% presented with a moderate or severe GCS, respectively. Chi-square analysis showed that GCS was affected by age (*p* = 0.003), such that the older patients were more likely to present with a mild GCS (Table 2). The CT Rotterdam Score was known for 1,969 (62%) patients. Linear regression showed that the relationship between GCS and the CT Rotterdam Score varied with age, such that older patients (>65 group) had a higher average GCS score (*p* = 0.027) compared to younger patients with the same CT Rotterdam Score. This separation in GCS from the oldest age group to the younger groups was more pronounced as the CT Rotterdam Score worsened (Figure 2).

**Table 2:** The effects of patient age and GCS on outcomes at 3 and 6 months following traumatic brain injury.

**Age group (years)**		**Unfavorable outcome at 3 months *n* (%)**	**Unfavorable outcome at 6 months *n* (%)**	**Odds ratio unfavorable outcome 3 months OR (95% CI)**	**Odds ratio unfavorable outcome 6 months OR (95% CI)**
All patients	18–34	228 (31)	181 (27)	**Reference**	**Reference**
	35–50	139 (32)	135 (31)	1.2 (0.9–1.6)	1.3 (1.0–1.6)
	51–65	258 (40)	213 (41)	1.5 (1.2–2.0)	1.8 (1.4–2.3)
	>65	555 (62)	508 (61)	3.7 (3.0–4.6)	4.2 (3.3–5.2)
Mild GCS	18–34	29 (7)	26 (6)	**Reference**	**Reference**
	35–50	37 (4)	34 (13)	2.9 (1.7–5.0)	2.3 (1.3–4.0)
	51–65	118 (27)	91 (16)	5.6 (3.4–9.2)	4.9 (3.0–8.1)
	>65	400 (56)	363 (55)	20.0 (12.6–31.6)	18.3 (11.5–28.9)
Moderate GCS	18–34	48 (36)	33 (27)	**Reference**	**Reference**
	35–50	25 (36)	27 (36)	1.3 (0.7–2.4)	1.6 (0.9–3.0)
	51–65	50 (55)	41 (60)	2.8 (1.5–5.3)	4.0 (2.1–7.6)
	>65	79 (86)	73 (81)	10.9 (5.4–22.3)	11.0 (5.7–21.4)
Severe GCS	18–34	151 (81)	122 (72)	**Reference**	**Reference**
	35–50	139 (78)	74 (74)	1.0 (0.5–1.8)	1.2 (0.7–2.0)
	51–65	258 (80)	81 (83)	0.9 (0.5–1.8)	2.0 (1.0–3.7)
	>65	555 (90)	72 (89)	2.0 (0.9–4.5)	3.2 (1.5–6.9)

*Unfavorable outcome was defined as upper severe disability, lower severe disability, vegetative, and dead as graded by the Extended Glasgow Outcome Score*.

### Outcome at 3 Months

GOSE at 3-months was known for 2,713 patients (85% of the entire cohort) and was significantly affected by categorical age (*p* < 0.001), such that the 51–65 age group was 1.5 (OR = 1.5, 95% CI 1.2–2.0) times more likely to have an unfavorable outcome when compared to the 18–34 age group (Table 2). The oldest age group (> 65 years old) had the largest risk of unfavorable outcome, being 3.7 (OR = 3.7, 95% CI 3.0–4.6) times more likely to have an unfavorable outcome when compared to the 18–34 age group (Table 2). In a logistic regression model with age as the continuous and sole explanatory variable, every year older increased the odds of an unfavorable outcome by 2.6% at 3 months (OR = 1.026, *p* < 0.001). When controlling for GCS and its interaction with age, there continued to be a significant effect (*p* < 0.001) on GOSE, with the relationship being more pronounced following mild and moderate injuries, while outcomes following severe injuries were similar across age groups (Table 2). The interaction between injury severity as measured by the CT Rotterdam Score and age was statistically significant (*p* < 0.0001) showed similar trends (Table 3 shows representative ORs at three different CT Rotterdam Scores).

**Table 3:** The effects of patient age and representative CT Rotterdam Scores on outcomes at 3 and 6 months following traumatic brain injury.

**CT Rotterdam Score**	**Age group (years)**	**Odds ratio unfavorable outcome 3 months OR (95% CI)**	**Odds ratio unfavorable outcome 6 months OR (95% CI)**
1	18–34	**Reference**	**Reference**
	35–50	1.7 (0.9–3.4)	2.9 (1.7–5.0)
	51–65	3.0 (1.6–5.7)	5.6 (3.4–9.2)
	>65	16.2 (9.1–28.7)	20.0 (12.6–31.6)
3	18–34	**Reference**	**Reference**
	35–50	1.4 (1.0–1.9)	1.3 (0.7–2.4)
	51–65	2.2 (1.7–3.0)	2.8 (1.5–5.3)
	>65	4.4 (3.7–5.8)	10.9 (5.4–22.3)
5	18–34	**Reference**	**Reference**
	35–50	1.1 (0.6–2.3)	0.9 (0.5–1.8)
	51–65	1.7 (0.8–3.4)	0.9 (0.5–1.8)
	>65	1.2 (0.6–2.3)	2.0 (0.9–4.5)

*Unfavorable outcome was defined as upper severe disability, lower severe disability, vegetative, and dead as graded by the Extended Glasgow Outcome Score*.

### Outcome at 6 Months

GOSE at 6-months was known for 2,464 patients (78% of the entire cohort). The relationship between age and outcome was similar to what was seen at 3-months, such that the 51–65 age group and > 65 age group had 1.8 (OR = 1.8, 95% CI 1.4–2.3) and 4.2 (OR = 4.2, 95% CI 3.32–5.2) times the likelihood of having an unfavorable outcome when compared to the 18–34 age group (Table 2), respectively. In a logistic regression model with age as the continuous and sole explanatory variable, every year older increased the relative odds of an unfavorable outcome by 2.9% at 6 months (OR = 1.029, *p* < 0.001). When controlling for GCS, age continued to have a significant effect (*p* = 0.03) on outcome, similar to the relationships seen at 3-months. When controlling for injury severity as measured by the CT Rotterdam Score, age continued to have a significant effect (*p* < 0.0001) on the GOSE at 6 months (Table 3). The change in outcome from three to 6 months was significantly affected by age (*p* < 0.001), such that the younger age groups were more likely to have improved overtime than the two older age groups, although the effect size was small (Table 2).

## Discussion

As the population of the United States continues to age, our understanding of the presentation and outcome following geriatric TBI will need to evolve. The data presented here confirms that in our medical center, the average age of patients suffering a TBI increased by 4.4 years from 2009 to 2016 and that patients older than 65 years were more frequently encountered than younger age groups. This institutional trend mirrors what is happening at the national level, where the incidence of TBI continues to increase in the elderly population, particularly in patients >75 years of age, while incidence in the younger population has remained relatively unchanged (10, 14, 15). In fact, TBI-related hospital visits among the oldest segment of the U.S. population has actually exceeded population growth in recent years (14). In line with the increasing age of the TBI population, fall was the most commonly encountered mechanism of injury in our cohort (39% of the population), followed by MVC (20%). An injury of the elderly, nearly 80% of patients who suffered a fall in our cohort were older than the age of 50 and is consistent with other published reports (14, 15, 19). This is consistent with the elderly populations increased risk for frailty and multiple co-morbidities (5, 7, 10).

Most of the patients in our cohort suffered a mild TBI, while much of the literature focuses on patients with moderate-to-severe injuries. In this study, the interaction between age group and GCS on admission was statistically significant, such that patients older than 65 years were more likely to present with a mild GCS. To our knowledge, this is only the second study that has compared the distribution of TBI severity across age categories. The first was an analysis of the National Trauma Data Bank National Sample Program TBI cases and did not find a difference in TBI severity across several age categories, with most patients presenting after mild injury (14). Differences in this study and ours may be related to sample size (522,882 vs. 3,179 patients) and age categories, with the previous study having smaller age ranges (10 year increments starting after the age of 45). Regardless of these study differences, it's clear that most elderly patients are presenting with a mild GCS, and, as we and others have shown, suffer worse outcomes.

The results of this analysis show that the relationship between age and unfavorable outcome at three- and 6-months post-injury is continuous. This has been previously been demonstrated after moderate and severe TBI, with some studies suggesting an inflection point toward worse outcomes beginning between ages 30 and 60 years (20). In our study, this relationship remains continuous when mild injuries are incorporated, with no inflection point being found. In particular, we found that the relative odds of an unfavorable outcome at 3- and 6-months increased by 2.6 and 2.9%, respectively, for every year older. This relationship was preserved when age was used as a categorical variable, with the two oldest age groups having significantly higher odds ratios for unfavorable outcome at both three- and 6-months post-injury when compared to the youngest age group.

The effect of age on outcome was most pronounced in patients who presented with a mild GCS; the effect was still statistically significant in patients with a moderate injury, but to a lesser extent. Surprisingly, many patients suffered an unfavorable outcome following a mild TBI, occurring in 6–16% of the youngest three age groups. This cause of such a high rate is not known, but could likely be related to uncontrolled confounders, such as the presence of polytrauma. In the oldest age group, 55% suffered an unfavorable outcome following a mild brain injury. The relationship between GCS and age is complex, with multiple studies showing that the elderly routinely present with a worse Abbreviated Injury Score of the Head (AIS) when compared to younger patients with the same GCS (14, 19, 21). While we were not able to retrospectively calculate AIS, we did find a statistically significant interaction effect between age and CT Rotterdam Scores on GCS, such that older patients presented with a better GCS than their younger counterparts with similar CT Rotterdam Scores. In addition, this effect of age was most prominent in patients with the worst CT Rotterdam Scores. Several theories have been postulated to explain this discrepancy between GCS and other measures of injury severity in the elderly. Salotolla et al. (21) postulate that the elderly patients have a blunted or delayed physiological response to trauma when compared to younger patients, which could include a lesser neuroinflammatory response or differences in vasoreactivity and cerebral edema formation. In addition, brain atrophy is common in the elderly, which may allow for expansion of mass lesions without significant neurological symptoms. In this scenario, the AIS or CT Rotterdam Score would overestimate injury severity secondary to a large mass lesion with little or no change in the GCS. However, analysis of our cohort suggests that CT Rotterdam Scores may underestimate injury severity, particularly in patients with lower CT Rotterdam Scores, as age had a much more profound effect on outcome when CT Rotterdam Scores were low. It is clear, then, that we do not have a definitive tool to understand and characterize TBI severity in the geriatric population. Given the elderly have worse outcomes even with better GCS scores at presentation, the admission GCS may not be an appropriate measure of injury severity. This has direct ramifications regarding patient care, as the geriatric patient may not receive the necessary acute or longitudinal interventions they need in light of a relatively high GCS. Further studies, including use of other severity scores like the AIS and CT Rotterdam Score, are necessary to develop predictive models of outcome in the vulnerable elderly population.

In patients who suffered a severe TBI, no statistically significant differences in outcome were seen among the four age groups at 3-months. However, by 6-months, outcomes were once again statistically worse in the two oldest age categories when compared to the youngest. It is well-established that elderly patients have slower rates of functional and cognitive recovery (15, 22) and this was seen in our population as whole, in which more patients from the youngest age group showed neurological improvement from 3–6-months than the other age groups. It is not entirely clear why elderly patients show slower recovery after TBI. Some have postulated a bias in clinical care resulting in slower acute interventions, higher likelihood to withdraw care, and increased likelihood to be discharged to a nursing facility instead of acute rehabilitation in light of poor rehabilitation tolerance (10, 14, 15, 22). It is also possible that our measurements of functional outcome do not accurately capture the functional status of the elderly population following TBI (15). While the GOSE is the most widely used measure of global functional outcome following TBI and has been recommended as the standard outcome measure for TBI studies, it has not been validated in the elderly population. One group showed continued improvement in functional outcome in the elderly up to 1-year following severe TBI using the Health Related Quality of Life Measure, an improvement that was not appreciated using the GOSE (23). In addition, the GOSE does not distinguish disability related to neurological impairment from that of systemic injury or illness; one could then postulate that in the setting of increased co-morbidities in the elderly, GOSE scores remain low in the elderly secondary to non-neurological conditions. Future work is necessary to validate functional outcome markers in the elderly.

### Strength and Limitations

The main strength of this study is the large sample size from a prospectively collected database from a busy Level 1 Trauma Center that manages all patients with TBI regardless of age, insurance status, or ability to pay. Accordingly, it is likely that our patient population better reflects the diversity of the TBI population as a whole than do clinical trials with strict inclusion and exclusion criteria.

A major limitation of this study includes the registry not containing all pertinent data, including data regarding patient comorbidities, presence and severity of systemic injuries, coagulopathy, surgical interventions, hospital complications, length of stay and post-discharge disposition, rehabilitation, and care. These uncontrolled confounding factors are known to contribute to patient outcome and further research is necessary to control for these factors across age groups. It is also possible that the mechanism of injury was not always accurately known by the treating team. We were not able to determine which patients transferred from UC Davis after presenting to a different hospital first, and therefore were not able to determine the effects of transfer time or measure any differential treatment strategies based on the patients' severity of injury or age. Patients who were lost to follow-up also introduce bias that we were not able to control for.

## Conclusion

The proportion of patients with TBI that are elderly is likely to continue to increase as the age of the population increases. Age is a major independent risk factor for unfavorable functional outcome when controlling for injury severity, both by radiographic and clinical severity scores. The largest disparity in outcomes across age was seen in patients who present with a mild GCS and CT Rotterdam Scores, suggesting that these markers of injury severity may underestimate the severity of injury in the elderly population. This information has clinically meaningful ramifications and highlights the need for clinical trials and validation of outcome markers in the elderly TBI population.

## Data Availability Statement

The datasets for this article are not publicly available because dissemination of data is not included in the study's IRB approval. Requests to access the datasets should be directed to Ryan Martin, MD at rymartin@ucdavis.edu.

## Ethics Statement

The studies involving human participants were reviewed and approved by UC Davis Interval Review Board. Written informed consent for participation was not required for this study in accordance with the national legislation and the institutional requirements.

## Author Contributions

NG, AT, and RM participated in the literature search and participated in data collection. NG, AT, MW, KS, and RM participated in the study design, participated in data interpretation, and participated in critical revision. NG, MW, and RM participated in data analysis and participated in writing.

### Conflict of Interest

The authors declare that the research was conducted in the absence of any commercial or financial relationships that could be construed as a potential conflict of interest.
